# A coordinate deregulation of microRNAs expressed in mucosa adjacent to tumor predicts relapse after resection in localized colon cancer

**DOI:** 10.1186/s12943-018-0770-8

**Published:** 2018-01-31

**Authors:** Angela Grassi, Lisa Perilli, Laura Albertoni, Sofia Tessarollo, Claudia Mescoli, Emanuele D. L. Urso, Matteo Fassan, Massimo Rugge, Paola Zanovello

**Affiliations:** 1 Istituto Oncologico Veneto IOV – IRCCS, Padova, Italy; 2 Department of Surgery, Oncology and Gastroenterology, University of Padova, Padova, Italy; 3 Surgical Pathology and Cytopathology Unit, Department of Medicine DIMED, University of Padova, Padova, Italy; 4 Azienda Ospedaliera-Università degli Studi di Padova, Padova, Italy

**Keywords:** microRNAs, localized colon cancer, relapse, predictive markers, adjacent mucosa

## Abstract

Up to 20% of colorectal cancer (CRC) node-negative patients develop loco-regional or distant recurrences within 5 years from surgery. No predictive biomarker able to identify the node-negative subjects at high risk of relapse after curative treatment is presently available.

Forty-eight localized (i.e. stage I-II) colon cancer patients who underwent radical tumor resection were considered. The expression of five miRNAs, involved in CRC progression, was investigated by qRT-PCR in both tumor tissue and matched normal colon mucosa.

Interestingly, we found that the coordinate deregulation of four miRNAs (i.e. miR-18a, miR-21, miR-182 and miR-183), evaluated in the normal mucosa adjacent to tumor, is predictive of relapse within 55 months from curative surgery.

Our results, if confirmed in independent studies, may help to identify high-risk patients who could benefit most from adjuvant therapy. Moreover, this work highlights the importance of extending the search for tissue biomarkers also to the tumor-adjacent mucosa.

**Electronic supplementary material:**

The online version of this article (10.1186/s12943-018-0770-8) contains supplementary material, which is available to authorized users.

## Background

Colorectal cancer (CRC) is the third most common tumor in the United States [1]. Up to 20% of node-negative (stage I and stage II) patients develop loco-regional or distant recurrences within 5 years from wide surgical resection and anastomosis. No predictive biomarker able to identify the node-negative subjects at high risk of relapse after curative surgery is presently available. Adjuvant chemotherapy is considered for stage II patients with at least one of the following clinical characteristics: sampling of fewer than 12 lymph nodes during surgical resection; poorly differentiated tumor; vascular, lymphatic or perineural invasion; tumor presentation with obstruction or tumor perforation and pT4 stage [2]. In this setting, the identification of biomarkers to guide adjuvant therapy decision could be extremely useful for clinical practice.

CRC develops and progresses through the accumulation of alterations affecting both noncoding and coding RNAs. Noncoding RNAs control multiple biological processes and have emerged as possible biomarkers for the clinical diagnosis and prognosis of CRC [3]. Different microRNA (miRNA) expression patterns observed in primary CRC have also been associated with tumor stage and patient survival [4].

In previous work, we identified up- and down-regulated miRNAs in primary colon carcinoma versus normal colon mucosa samples from metastatic CRC patients and reconstructed putative post-transcriptional regulatory sub-networks involving the most differentially expressed miRNAs and their target genes [5].

More recently, a study by Sanz-Pamplona and colleagues demonstrated that a number of tumor-related genes were activated in the tumor-adjacent non-neoplastic mucosa of CRC patients. These activated genes were enriched in transcription factors, indicating the existence of a transcriptional program driving the observed altered expression pattern in normal mucosa [6]. This result paves the way for the possibility to detect tumor-specific alterations also in the adjacent mucosa.

The focus of this paper is to identify miRNAs that could serve as biomarkers of relapse after resection in localized colon cancer, by investigating both matched normal colon mucosa and tumor tissue, resected from the surgical specimen.

## Findings

In this study we explored the usefulness of a set of five miRNAs (i.e. miR-18a, miR-21, miR-182, miR-183 and miR-139) as biomarkers of relapse after primary tumor resection in stage I-II colon cancer.

These miRNAs were already known to be involved in CRC progression. In particular, miR-21 is a known oncomiR, whose high expression levels have been related to inflammation-associated colorectal tumorigenesis and dismal clinical outcome [7]. MiR-18a was found to be significantly up-regulated in patients with a form of chronic inflammation, i.e. colonic polyps [8], and it has been suggested as a prognostic factor for survival of post-operative CRC patients [9]. The increased expression of miR-183 and miR-182, both members of miR-183 cluster, has been related to advanced clinical stage, lymph node involvement, distant metastases and poor prognosis [10, 11]. Concerning miR-139, a recent study highlighted its down-regulation in colon cancer cell lines and patients and its association with metastasis and drug resistance [12].

### Differentially expressed microRNAs in localized colon cancer between tumor and matched normal mucosa

**Fig. 1:**
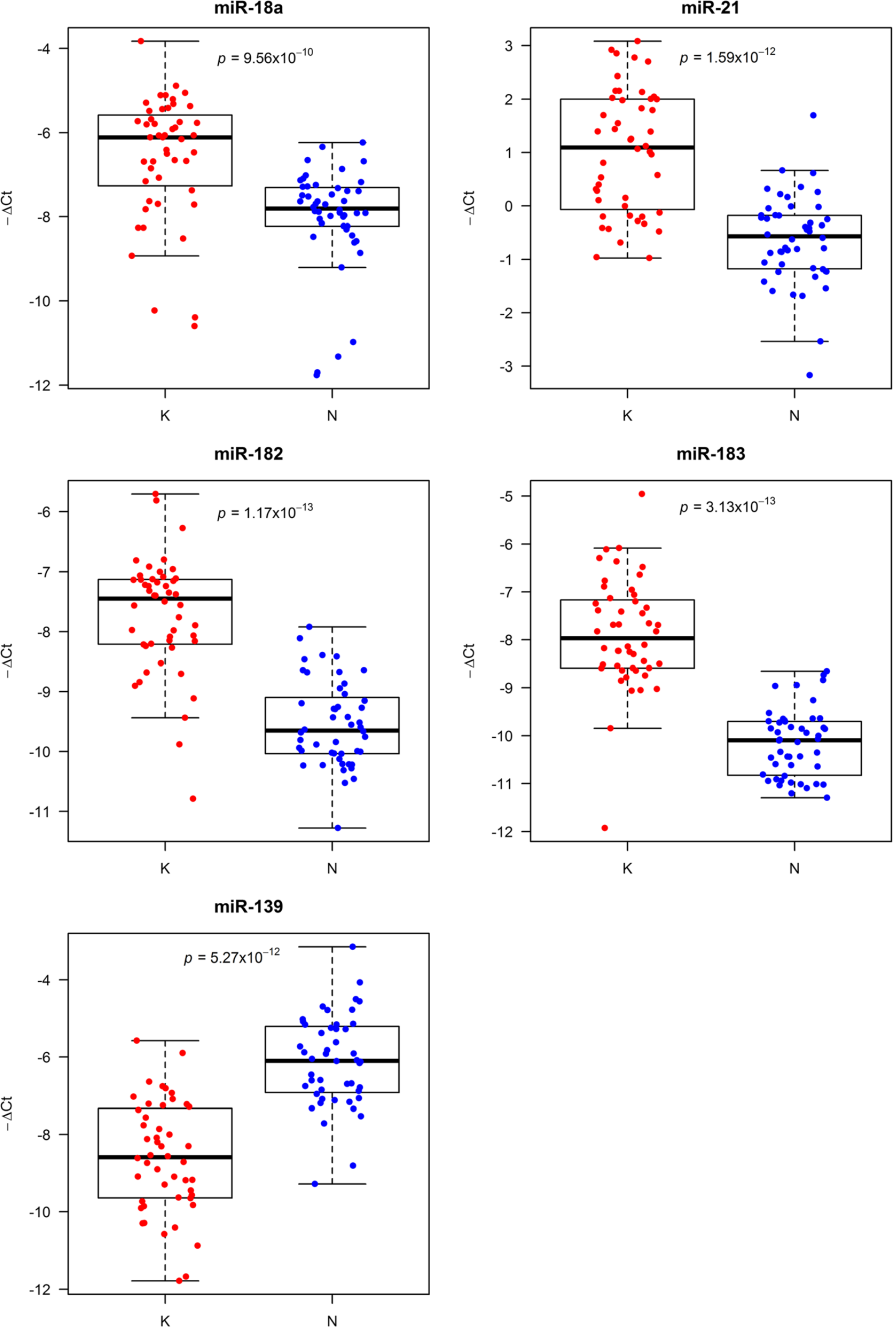
Boxplots of the distribution of –ΔCt values in tumor tissue versus matched normal mucosa for miR-18a, miR-21, miR-182, miR-183 and miR-139. Each dot represents a patient sample. –ΔCt values were calculated using miR-200c as a reference. Differences between cancer tissue (K) and matched normal mucosa (N) samples were analyzed using one-tailed Wilcoxon rank-sum test

Forty-eight patients with sporadic stage I-II colon cancer were considered and both tumor tissue and matched normal mucosa were collected at the time of curative surgery, see Additional file 1. The expression levels of the five selected miRNAs, already known to be modulated in advanced CRC (stage IV), were evaluated. Interestingly, our analysis confirmed that they were all significantly regulated in the early phases of the CRC tumor process. Specifically, miR-18a, miR-21, miR-182 and miR-183 were strongly up-regulated (*p* < < 0.001) in cancer tissue with respect to normal mucosa, whereas miR-139 was strongly down-regulated (*p* < < 0.001), see Fig. 1. This finding extends to stage I-II CRC the results previously obtained in stage IV CRC [5], showing that these five miRNAs accompany the CRC tumor process, from initial stages to advanced tumorigenesis.

To assess the role of tumor-associated inflammation in the modulation of these miRNAs, we also evaluated their expression levels in 10 patients affected by ulcerative colitis, a form of inflammatory bowel disease characterized by chronic and widespread inflammation of the colorectal mucosa. MiR-18a and miR-21 appeared weakly up-regulated in the inflammatory tissue vs. normal mucosa with *p*-value of 0.053 and 0.042, respectively. Conversely, miR-182, miR-183 and miR-139 were not significantly modulated (Additional file 1: Fig. S1). Although with a limited sample size, our data indicate that the modulations of miR-182, miR-183 and miR-139 are more specific of the tumor process. MiR-18a and miR-21, instead, appear weakly up-regulated also in inflamed bowel tissue, in line with previously published works [7, 8], thus suggesting that the strong up-regulation of these two miRNAs in the tumor tissue could be partially related to inflammation.

### miRNA ratios as possible biomarkers of relapse

Based on recurrence-free survival (RFS), the 48 localized colon cancer patients in study were subdivided into a recurrent group (R, 23 patients with RFS > 55) and a non-recurrent group (NR, 25 patients with RFS < 55), see Additional file 1. The clinical characteristics of the patients are described in Table 1.

**Table 1:** Clinical characteristics of relapsing (R) and non-relapsing (NR) patients

Characteristics		R	NR
		*n* = 23	*n* = 25
Age at resection (years)	Median	72	69
	Range	55–85	50–90
Sex	M	16 (70%)	10 (40%)
	F	7 (30%)	15 (60%)
Tumor site	Cecum, colon ascending, hepatic (right) flexure	7	6
	transverse colon	3	4
	Splenic (left) flexure, colon descending, sigmoid colon	13	15
	Rectum	0	0
TNM stage	I	6	7
	II	17	18
T(n)	T1	2	1
	T2	4	6
	T3	15	18
	T4	2	0
N(n)	N0	23(100%)	25(100%)
M(n)	M0	23 (100%)	25 (100%)
Grading (n)	G1	5	4
	G2	15	17
	G3	3	4
	G4	0	0

**Fig. 2:**
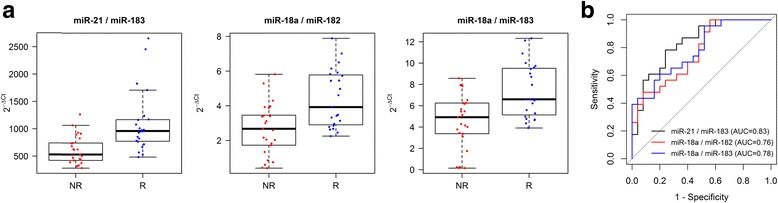
miR-21/miR-183, miR-18a/182 and miR-18a/183 ratios in tumor-adjacent mucosa predict relapse of colon cancer after bowel resection. **a**. The three panels show the distribution of miRNA ratio relative expression levels, indicated as 2^-ΔCt^, in localized colon cancer patients, relapsing (R) and non-relapsing (NR) within 55 months after resection. **b**. ROC curves generated for the three significant miRNA ratios (*p* < 0.01) in univariate logistic regression. Corresponding areas under ROC curves (AUC) are detailed in the figure

To investigate whether the selected miRNAs could be useful to predict tumor relapse, we applied the miRNA ratio approach [13, 14]. We calculated 10 ratios between the expression values of all possible miRNA pair combinations (see Additional file 1) in both the tumor tissue and the adjacent normal mucosa, and assessed their capability to predict relapse through univariate logistic regression analysis, Additional file 1: Table S1. None of the miRNA ratios resulted predictive when evaluated in the colon cancer tissue. Three miRNA ratios, evaluated in the tumor-adjacent mucosa, were found to be significant predictors of relapse within 55 months from resection: miR-21/miR-183 (*p* = 0.0011), miR-18a/miR-182 (*p* = 0.0053) and miR-18a/miR-183 (*p* = 0.0099), see Fig. 2a and Additional file 1: Table S2 for details about univariate logistic regression models. Corresponding areas under ROC curves were 0.83, 0.76 and 0.78, respectively, see Fig. 2b. It is noteworthy that the three significant miRNA ratios (miR-21/miR-183, miR-18a/miR-182 and miR-18a/miR-183) together with miR-21/miR-182 ratio, which appears weakly significant (*p* = 0.063, Additional file 1: Table S1), share a common characteristic. These miRNA ratios have at numerator a microRNA that in our setting appears associated also to inflammation (either miR-18a or miR-21) and at denominator a microRNA more specific of the tumor (either miR-183 or miR-182). We thus conclude that a coordinate deregulation of these two couples of miRNAs may be a useful predictor of relapse. To investigate how the individual miRNAs perform as predictive biomarkers, an additional univariate logistic regression analysis was executed and reported in Additional file 1: Table S3, showing that the miRNA ratio approach yields to more significant results. Moreover, a bivariate logistic regression analysis, taking as covariates all the possible combinations of miRNA pairs, was conducted (Additional file 1: Table S4). This analysis confirmed that, regardless of the chosen approach, it is the coordinated alteration of the two couples of miRNAs that we have identified to be predictive of relapse. The advantage of the miRNA ratio approach is to overcome the need for miR-200c as a normalizer.

More interestingly, the three significant miRNA ratios seem to be potential biomarkers when evaluated in the adjacent, morphologically normal, mucosa and not in the tumor tissue. This result, apparently counterintuitive, is in line with recent gene expression studies on the adjacent mucosa conducted both in CRC [6] and in head and neck cancer [15]. Indeed, it is emerging with increasing evidence that the crosstalk between tumor and microenvironment could also affect the adjacent mucosa and that also this tissue may be informative.

## Conclusion

Our analysis showed that not a single miRNA, but rather a coordinated alteration of four miRNAs (i.e. miR-18a, miR-21, miR-182 and miR-183) may be useful to predict recurrence after curative surgery. This is the first study that outlines a predictive role of miRNAs, evaluated in the adjacent, morphologically normal, mucosa of CRCs. Our results, if confirmed in an ample cohort of patients, may help to identify among localized colon cancer (stage I-II) patients those at high risk of relapse who could benefit most from adjuvant therapy.

## Additional file

Additional file 1: Supplementary information including Materials and Methods, **Tables S1-S4** and **Figure S1**. (PDF 157 kb)

## Abbreviations

AUC: Area under ROC curve
CRC: Colorectal cancer
miRNA: microRNA
RFS: Recurrence-free survival
