# Supplementary material for: A coordinate deregulation of microRNAs expressed in mucosa adjacent to tumor predicts relapse after resection in localized colon cancer
Source: Mol Cancer. 2018 Jan 31;17:17. doi: 10.1186/s12943-018-0770-8 (PMC5791208; doi:10.1186/s12943-018-0770-8)

SUPPLEMENTARY MATERIALS AND METHODS

Patients. Forty-eight patients with sporadic localized colon adenocarcinomas, who underwent radical surgical treatment between January 2003 and October 2008, were selected from the institutional database. The study was approved by the Ethics Committee of the University Hospital of Padua (n. 57841 December 3rd 2013) and informed consent was obtained from all the patients involved. Pathological cancer staging was done according to the 7th Edition of the Tumor, Node, Metastasis (TNM) classification for colorectal cancer. All patients were at TNM stages I or II, with no regional lymph node metastases (N0) and no distant metastases (M0), and none of them received neoadjuvant or adjuvant therapy.

Recurrence-free survival (RFS) was defined as the length of time from radical primary tumor resection until the detection of loco-regional or distant recurrence or decease due to any cause.

Patients were thus subdivided into a Recurrent (R) group, 23 patients with RFS less than 55 months, and a Non-Recurrent (NR) group, 25 patients with RFS greater than 55 months. Recurrent disease was prevalently observed at distant site (i.e. 8 hepatic, 5 pulmonary, 1 peritoneal, 1 lymph nodal and 7 not specified metastases) with only one local recurrence (anastomotic). The clinical characteristics of these patients are reported in the main manuscript, see Table 1.

Finally, 10 patients affected by a form of Inflammatory Bowel Disease (IBD), in particular moderate ulcerative colitis, were independently evaluated.

Histopathological FFPE samples. Original slides or serial sections (4–6 µm thick) obtained from archival paraffin-embedded tissue samples (H&E) were jointly re-assessed by two expert gastrointestinal pathologists (MF and MR) according to current criteria (WHO 2010). For each patient, a sample of CRC-adjacent, morphologically normal colon mucosa was dissected from the proximal tumor resection margin, with a minimum distance of 3 centimeters from the primary tumor.

RNA isolation and quantitative RT-PCR. Hematoxylin and eosin stained sections of each specimen were prepared and evaluated, and only samples with more than 70% of vital tumor tissue were considered for RNA extraction. Total RNA was isolated from FFPE (Formalin Fixed Paraffin Embedded) samples using the RecoverAll Total Nucleic Acid Isolation Kit (Ambion, Austin, TX) according to the manufacturer's instructions. The concentration of RNA was quantified by NanoDrop 1000 Spectrophotometer (NanoDrop Technologies, Waltham, MA).

Total RNA was used for first-strand cDNA synthesis in a 15µl reaction volume, using the TaqMan miRNA Reverse Transcription kit and miRNA-specific stem-loop primers (Thermo Fisher Scientific, Foster City, CA, USA). We conducted qRT-PCR experiments amplifying cDNA for 45 cycles using TaqMan miRNA primers and probes (Thermo Fisher Scientific) and LightCycler 480 PCR Master Mix (Roche Diagnostics, Mannheim, Germany). All reactions were performed in triplicates, including no template controls, using LightCycler 480 II Real-Time System (Roche Diagnostics).

Data normalization and statistical analysis

RNU44 and miR-200c were tested as candidate normalizers. MiR-200c, already identified as most stable miRNA in metastatic CRC [1], was also confirmed as best normalizer in localized CRC. Relative expression of target miRNAs was calculated as $\Delta Ct_{miR} = Ct_{miR} - Ct_{normalizer}$.

To apply the miRNA ratio approach [2, 3], the Ct value of each miRNA was converted into the corresponding expression level (2^{-Ct}).

Then the miRNA ratios between all possible miRNA pair combinations (e.g. miR-x/miR-y ratio) were calculated as $2^{-\Delta Ct} = 2^{-(Ct_{miR-x} - Ct_{miR-y})}$. It is to be noted that only one ratio was considered for each miRNA pair (e.g. miR-x/miR-y) and not the reciprocal (miR-y/miR-x).

Statistical analysis. A one-tailed Wilcoxon rank-sum test was used to identify miRNAs significantly different between matched CRC tissue and adjacent normal mucosa. A univariate

logistic regression model was built to evaluate the ability of each miRNA ratio on log_2-scale to predict the relapse within 55 months. Odds ratios and 95% confidence intervals were estimated for the fitted logistic regression models. Receiver operating characteristic (ROC) curves were plotted and the area under the ROC curve (AUC) was estimated to compare the most significant miRNA ratios. Statistical analyses were performed in the R environment using a customized code and the pROC package for ROC curve analysis.

Supplementary References

1. Pizzini S, Bisognin A, Mandruzzato S, Biasiolo M, Facciolli A, Perilli L, et al. Impact of microRNAs on regulatory networks and pathways in human colorectal carcinogenesis and development of metastasis. BMC Genomics 2013;14:589.

2. Boeri M, Verri C, Conte D, Roz L, Modena P, Facchinetti F, et al. MicroRNA signatures in tissues and plasma predict development and prognosis of computed tomography detected lung cancer. Proc Natl Acad Sci U S A 2011;108(9):3713-8.

3. Sharova E, Grassi A, Marcer A, Ruggero K, Pinto F, Bassi P, et al. A circulating miRNA assay as a first-line test for prostate cancer screening. Br J Cancer 2016;114(12):1362-6.

SUPPLEMENTARY TABLES AND FIGURES

Table S1. Evaluation of capability of predicting relapse of miRNA ratios in normal mucosa adjacent to tumor and in tumor tissue.

A univariate logistic regression model was developed for each miRNA ratio to evaluate its capability to distinguish between patients who were relapsing within 55 months after bowel resection and those who were not, both in normal mucosa adjacent to tumor and in tumor tissue. The corresponding area under the ROC curve was calculated and reported in table as AUC. Three miRNA ratios resulted significant predictors of relapse (p<0.01 and AUC>0.75) in normal mucosa adjacent to tumor.

	NORMAL MUCOSA		TUMOR TISSUE	
**miR_ratio**	**p-value**	**AUC**	**p-value**	**AUC**
miR-18a/miR-21	0.3084	0.58	0.40	0.51
**miR-18a/miR-182**	**0.0053**	**0.76**	0.46	0.56
**miR-18a/miR-183**	**0.0100**	**0.78**	0.46	0.49
miR-18a/miR-139	0.1178	0.60	0.51	0.61
miR-21/miR-182	0.0632	0.69	0.79	0.49
**miR-21/miR-183**	**0.0011**	**0.83**	0.99	0.53
miR-21/miR-139	0.1794	0.66	0.08	0.67
miR-182/miR-183	0.1752	0.56	0.65	0.62
miR-182/miR-139	0.7342	0.53	0.23	0.62
miR-183/miR-139	0.1919	0.58	0.20	0.63

Table S2. Univariate logistic regression models for miR-21/miR-183, miR-18a/miR-182, miR-18a/miR-183.

The estimated coefficients with the corresponding standard errors (SE), the significance expressed as p-value (P), and odds ratios (OR) along with 95% confidence interval (CI) are reported for the three univariate logistic regression models. The estimated constant terms for the three models are also shown.

	Coefficient	SE	p-value	OR	95% CI
**miR-21/miR-183**	2.38	0.73	0.0011	10.771	3.122-56.913
**Constant**	-22.75	6.97	0.0011		
**miR-18a/miR-182**	1.65	0.59	0.0053	5.232	1.949-20.270
**Constant**	-2.85	1.10	0.0095		
**miR-18a/miR-183**	1.84	0.71	0.0100	6.300	1.875-30.589
**Constant**	-4.65	1.87	0.0129		

Table S3. Evaluation of capability of predicting relapse of individual miRNAs in normal mucosa adjacent to tumor and in tumor tissue.

A univariate logistic regression model was developed for each miRNA to evaluate its capability to distinguish between patients who were relapsing within 55 months after bowel resection and those who were not, both in normal mucosa adjacent to tumor and in tumor tissue. The corresponding area under the ROC curve was calculated and reported in table as AUC. In normal mucosa adjacent to tumor, miR-18a resulted significant ($p<0.05$) and miR-21 and miR-183 weakly significant ($p<0.10$) as predictors of relapse. In tumor tissue, only miR-139 was significant ($p<0.05$).

	NORMAL MUCOSA		TUMOR TISSUE	
miR	p-value	AUC	p-value	AUC
miR-18a	**0.031**	0.71	0.201	0.59
miR-21	**0.055**	0.71	0.655	0.53
miR-182	0.830	0.51	0.365	0.54
miR-183	**0.098**	0.64	0.670	0.52
miR-139	0.797	0.50	**0.038**	0.69

Table S4. Evaluation of capability of predicting relapse of miRNA pair combinations in normal mucosa adjacent to tumor.

A bivariate logistic regression model was developed for each miRNA pair to evaluate its capability to distinguish between patients who were relapsing within 55 months after bowel resection and those who were not. The two covariates, or independent variables, in the logistic regression model are denoted with C1 and C2 (e.g. for the model in the first row miR-18a is C1 and miR-21 is C2). The significance of each covariate in the model is reported in the corresponding p-value column. The area under the ROC curve was indicated as AUC. Only for three miRNA combinations (miR-18a + miR-182, miR-18a + miR-183, miR-21 + miR-183), both miRNAs resulted significant predictors of relapse (p<0.01) and AUC was greater than 0.75.

miR combination (C1 + C2)	p-value (C1)	p-value (C2)	AUC
miR-18a + miR-21	0.039	0.122	0.76
**miR-18a + miR-182**	**0.008**	**0.029**	**0.77**
**miR-18a + miR-183**	**0.011**	**0.030**	**0.78**
miR-18a + miR-139	0.038	0.982	0.70
miR-21 + miR-182	0.043	0.468	0.70
**miR-21 + miR-183**	**0.002**	**0.002**	**0.83**
miR-21 + miR-139	0.033	0.299	0.67
miR-182 + miR-183	0.457	0.089	0.62
miR-182 + miR-139	0.818	0.787	0.53
miR-183 + miR-139	0.058	0.319	0.65

Figure S1. Boxplots of the distribution of –ΔCt values in inflamed tissue versus matched normal mucosa for miR-18a, miR-21, miR-182, miR-183 and miR-139.

Each dot represents a patient sample. –ΔCt values were calculated using RNU44 as a reference, which was proven to be more stable than miR-200c in these samples. Differences between inflamed bowel tissue (F) and matched normal mucosa (N) from patients affected by moderate ulcerative colitis were analyzed using one-tailed Wilcoxon rank-sum test.

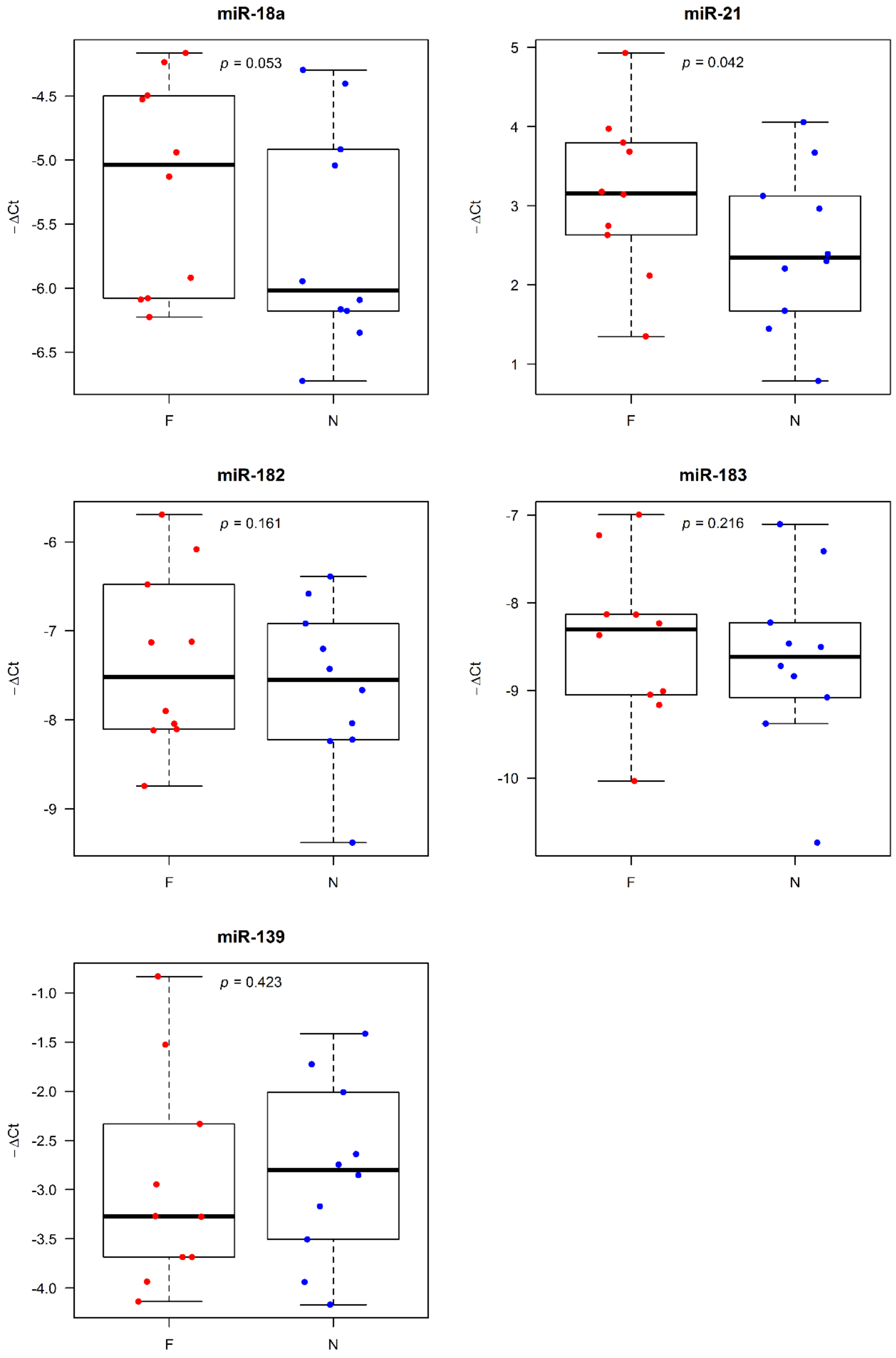

Supplement: Additional file 1: — Supplementary information including Materials and Methods, Tables S1-S4 and Figure S1. (PDF 157 kb) [file 12943_2018_770_MOESM1_ESM.pdf]
